# Smart Wireless Acoustic Sensor Network Design for Noise Monitoring in Smart Cities

**DOI:** 10.3390/s20174765

**Published:** 2020-08-23

**Authors:** Rosa Ma Alsina-Pagès, Patrizia Bellucci, Giovanni Zambon

**Affiliations:** 1 GTM–Grup de recerca en Tecnologies Mèdia, La Salle–Universitat Ramon Llull. c/Quatre Camins, 30, 08022 Barcelona, Spain; 2 ANAS S.p.A., DIV Research and Development. Via Monzambano, 10-00185 Rome, Italy; p.bellucci@stradeanas.it; 3 Department of Earth and Environmental Sciences (DISAT), Universitá degli Studi di Milano Bicocca, Piazza della Scienza 1, 20126 Milano, Italy; giovanni.zambon@unimib.it

**Keywords:** smart cities, noise monitoring, acoustic sensor design, noise mapping, acoustic event detection, map generation, public information, END, CNOSSOS-EU

## Abstract

This Special Issue is focused on all the technologies necessary for the development of an efficient wireless acoustic sensor network, from the first stages of its design to the tests conducted during deployment; its final performance; and possible subsequent implications for authorities in terms of the definition of policies. This Special Issue collects the contributions of several LIFE and H2020 projects aimed at the design and implementation of intelligent acoustic sensor networks, with a focus on the publication of good practices for the design and deployment of intelligent networks in any locations.

## 1. Introduction

The Environmental Noise Directive (END) requires that a five-year updating of noise maps be carried out, and requires checking and reporting on the changes that occurred during the reference period. The updating process is usually achieved using a standardized approach, consisting of collecting and processing information through acoustic models to produce the updated noise maps. This procedure is time consuming and costly, and has a significant impact on the financial statement of the authorities responsible for providing the maps. Furthermore, the END requires that easy-to-read noise maps are made available to the public, to provide information on noise levels, and that subsequent actions be undertaken by local and central authorities to reduce noise impacts.

In order to update the noise maps more easily and in a more effective way, it is convenient to design an integrated system incorporating real-time noise measurement and signal processing to identify and analyze the noise sources present in the mapping area (road traffic noise, leisure noise, etc.), and to automatically generate and present the corresponding noise maps. This wireless acoustic sensor network design requires transversal knowledge, from accurate hardware design for the acoustic sensors, to network structure design and management of the information, signal processing to identify the origin of the measured noise and graphical user interface application design to present the results to end users.

## 2. Contributions

This Special Issue presents eleven outstanding papers covering all the technologies necessary for the development of an efficient wireless acoustic sensor network, from the first stages of its design to the tests conducted during deployment; its final performance; and possible subsequent implications for authorities in terms of the definition of policies.

Picaut et al. [1] proposed an extensive review of the literature around low cost sensors for urban noise monitoring. Furthermore, they also identified the expected technical characteristics of the sensors to address the problem of noise pollution assessment. Finally, the paper also presents the challenges required to respond to a massive deployment of low-cost noise sensors.

Other authors have centered their work on the sensor network and node design. In Prieto et al. [2], the authors describe the design and implementation of a complete system for a WASN deployed in the city of Linares (Jaén-Spain). The complete system covers the network topology design, and the hardware and software of the sensor nodes elaboration, along with protocols and the web server platform. They provide several metrics about the noise measured in the nodes: $L_{Aeq}$ for a given period of time; $L_{den}$, $L_{day}$, $L_{evening}$ and $L_{night}$; the percentile noise levels (LA01T, LA10T, LA50T, LA90T and LA99T); the temporal evolution representation of noise levels; and the predominant frequency noise. López et al. [3] present a design for a versatile electronic device to measure outdoor noise, designed according to the technical standards of this type of instrument, and it has been tested following the regulations of the calibration laboratories for sound level meters (SLM). The evaluation of the quality of the electronics and the algorithm fully fit the requirements of a type 1 noise measurement instrument. Nevertheless, the use of an electret microphone reduces the technical features of the designed instrument into a type 2 noise measurement instrument. The authors deployed a low-cost sub-network in the city of Málaga (Spain) to analyze the leisure noise. The designed equipment is a two channel instrument, measuring simultaneously 86 sound parameters for each channel, such as $L_{eq}$ (with Z, C and A frequency weighting); the peak level (with Z, C and A frequency weighting); the maximum and minimum levels (with Z, C and A frequency weighting); the impulse, fast and slow time weightings; seven percentiles (1%, 5%, 10%, 50%, 90%, 95% and 99%); and continuous equivalent sound pressure levels in the one-third octave and octave frequency bands.

In terms of instrumentation, a contribution by Bianco et al. [4] studied the development of a measurement instrument that can be fastened by means of holding elements to a moving laboratory, a vehicle, for example. This device overcomes the fact that the measurements are usually on-site, and allows one to perform a continuous spatial characterization of a given pavement in order to yield a direct evaluation of the surface’s equality. The system was uncoupled from the vehicle by means of PID controller, evaluated to maintain the system at a fixed distance from the ground and reduced damping. Related to vehicles also, Flor et al. [5] focused on the evaluation of the noise level inside a vehicle by means of statistical tools. They made an experimental setup of microphones and a microcomputer strategically located on the car’s panel, and they conducted measurements under different conditions, including car window positions, rain, traffic and various speeds. They discussed the relevance of the variables that contribute to the noise level inside a car.

There are several papers about the analysis of the noise data gathered by means of wireless acoustic sensor networks. In Alías et al. [6], the authors took as a starting point the LIFE DYNAMAP project that developed a WASN-based dynamic noise mapping system based solely on road traffic noise (RTN) levels. After analyzing the bias caused by individual anomalous noise events (ANEs) on the computation of the A-weighted $L_{eq}$, they evaluated the aggregate impact of the ANEs on the RTN measurements in a real-operation environment. They evaluated this metric over more than 300 h of labeled acoustic data collected by means of two WASNs deployed in the project in the suburban area of Rome and in the urban area of Milan. Benocci et al. [7] also took as a starting point the LIFE DYNAMAP project, and their contribution was the final assessment of the system installed in the area of Milan. The traffic noise data gathered by the nodes, each one of them representative of a number of roads sharing the same characteristics, were used to build-up a real-time noise map. In particular, the analysis focused on the 21 sites belonging to the testing campaign. That allowed them to evaluate the accuracy and reliability of the system by comparing the predicted noise level of the DYNAMAP sensors with field measurements in several randomly selected sites.

Brambilla et al. [8] dealt in their study with the application of the intermittency ratio (IR) to urban road traffic noise data, collected in terms of 1-sec A-weighted sound pressure level (SPL), without any technician attending the measurements, and with continuous monitoring for 24 h in 90 different sites in the city of Milan. They show the IR computed for each hourly dataset of the 251 time series available—24 h each—including different types of roads, from motorways to local roads with low traffic flow. The authors processed the IR using clustering methods to extract the most significant temporal pattern features of IR to propose a criterion to classify the urban sites while taking into account road traffic noise events, which potentially increase the noise annoyance. Guarnaccia, in [9], presented EAgLE, which stands for equivalent acoustic level estimator. It is a new methodology, based on video processing and object detection tools. When the vehicles, their typology and their speed, are recorded, the sound power level of each vehicle is computed according to the EU recommended standard model CNOSSOS-EU [10] and the sound exposure level (SEL) of transit is estimated at the receiver. The $L_{eq}$ is evaluated in the end, summing up the contributions of all the vehicles.

Finally, in the Special Issue there are two contributions based on the prediction and the use of artificial intelligence on acoustic data. Navarro et al. [11] proposed the forecasting of temporal short-term sound levels as a useful tool for urban planners and managers. A long short-term memory (LSTM) deep neural network technique models the temporal behavior of sound levels at a certain location—both SPL and loudness level—in order to predict near-time future values. It can be trained and integrated for every node of a network to provide functionalities as a method of early warning against noise pollution, but also as a backup in case of a single node malfunction. The last contribution, by Glowacz [12], presents a fault detection technique of an electric impact drill (EID), coffee grinder A (CG-A) and coffee grinder B (CG-B) by means of acoustic signals. The three of them use commutator motors; the measurements of acoustic signals were carried out with a microphone. Seven signals from EID were measured, four of CG-A and three of CG-B, and an acoustic analysis was carried out, with good results in efficiency of recognition of all classes.

## Abbreviations

The following abbreviations are used in this manuscript:

ANE	Anomalous Noise Events
CNOSSOS-EU	Common Noise Assessment Methods in Europe
CG-A	Coffee Grinder A
CG-B	Coffee Grinder B
EID	Electric Impact Drill
END	Environmental Noise Directive 2002/49/EC
IR	Intermittency Ratio
LSTM	Long Short-Term Memory
RTN	Road Traffic Noise
SEL	Sound Exposure Level
SLM	Sound Level Meters
SPL	Sound Pressure Level
WASN	Wireless Acoustic Sensor Networks
